# Protein-Assisted Room-Temperature Assembly of Rigid, Immobile Holliday Junctions and Hierarchical DNA Nanostructures

**DOI:** 10.3390/molecules25215099

**Published:** 2020-11-03

**Authors:** Saminathan Ramakrishnan, Sivaraman Subramaniam, Charlotte Kielar, Guido Grundmeier, A. Francis Stewart, Adrian Keller

**Affiliations:** 1 Technical and Macromolecular Chemistry, Paderborn University, Warburger Str. 100, 33098 Paderborn, Germany; saminathan.ramakrishnan@gmail.com (S.R.); kielar86@hzdr.de (C.K.); guido.grundmeier@uni-paderborn.de (G.G.); 2 Structural Biophysics Laboratory, Center for Cancer Research, National Cancer Institute, Frederick, MD 21702, USA; 3 Biotechnology Center, Department of Genomics, Technische Universität Dresden, Tatzberg 47-51, 01307 Dresden, Germany; sivaraman.subramaniam@tu-dresden.de (S.S.); francis.stewart@tu-dresden.de (A.F.S.); 4 Cluster of Excellence Physics of Life, Technische Universität Dresden, 01062 Dresden, Germany

**Keywords:** DNA nanotechnology, Holliday junctions, atomic force microscopy, single-strand annealing proteins, Redβ

## Abstract

Immobile Holliday junctions represent not only the most fundamental building block of structural DNA nanotechnology but are also of tremendous importance for the in vitro investigation of genetic recombination and epigenetics. Here, we present a detailed study on the room-temperature assembly of immobile Holliday junctions with the help of the single-strand annealing protein Redβ. Individual DNA single strands are initially coated with protein monomers and subsequently hybridized to form a rigid blunt-ended four-arm junction. We investigate the efficiency of this approach for different DNA/protein ratios, as well as for different DNA sequence lengths. Furthermore, we also evaluate the potential of Redβ to anneal sticky-end modified Holliday junctions into hierarchical assemblies. We demonstrate the Redβ-mediated annealing of Holliday junction dimers, multimers, and extended networks several microns in size. While these hybrid DNA–protein nanostructures may find applications in the crystallization of DNA–protein complexes, our work shows the great potential of Redβ to aid in the synthesis of functional DNA nanostructures under mild reaction conditions.

## 1. Introduction

The Holliday junction (HJ) is an intermediate DNA structure involved in genetic recombination and DNA repair. The biological significance of HJs includes facilitating the strong binding of regulatory proteins and target sites of recognition signals during replication [1]. The folding or emergence of double helical junctions and their stability strongly rely on three critical factors: base stacking [2], sequence composition [3], and ionic strength [4]. However, innate branch migration in HJs is a distinct phenomenon that makes the HJ structures highly unstable [5]. Active pair exchange between bases in homologous strands and in particular at the branch points migrates the sequences along the structure until the HJ eventually disappears [6]. For previous structural and functional analyses, the in vitro production of HJs was required. Thereby, it became possible to understand how junction-resolving enzymes such as resolvases bind and split the duplexes in the HJ structures, which is an essential event in recombination [7]. Furthermore, the synthesis of immobile HJs is a cornerstone of structural DNA nanotechnology [8]. Initially, Seeman designed immobile HJ structures by reevaluating the concept of branch point migration and its influence on junction instability with the aim to arrange them in three-dimensional crystal lattices. [9]. Seeman et al. also studied the orientation-specific cleavage function of resolvase enzymes in synthetic HJ structures [10] and other critical biophysical characteristics [11,12,13,14,15]. The immediate applications of such synthetic HJs also included the understanding of functional proteins [16] and epigenetic markers [17], as well as applications in biomolecular engineering [18]. Importantly, in DNA nanotechnology, the concept of immobile HJ synthesis linked to a peculiar sticky-end strategy was employed to assemble highly ordered crystal-like lattices in two and three dimensions. Unfortunately, this strategy usually requires complex and time-consuming thermal annealing protocols, which in extreme cases may extend over several days [19], while typically resulting in only moderate synthesis yields. Consequently, there is a need for fast and low-temperature strategies for DNA nanostructure synthesis with high assembly yields [20,21,22,23,24,25,26].

**Figure 1:**
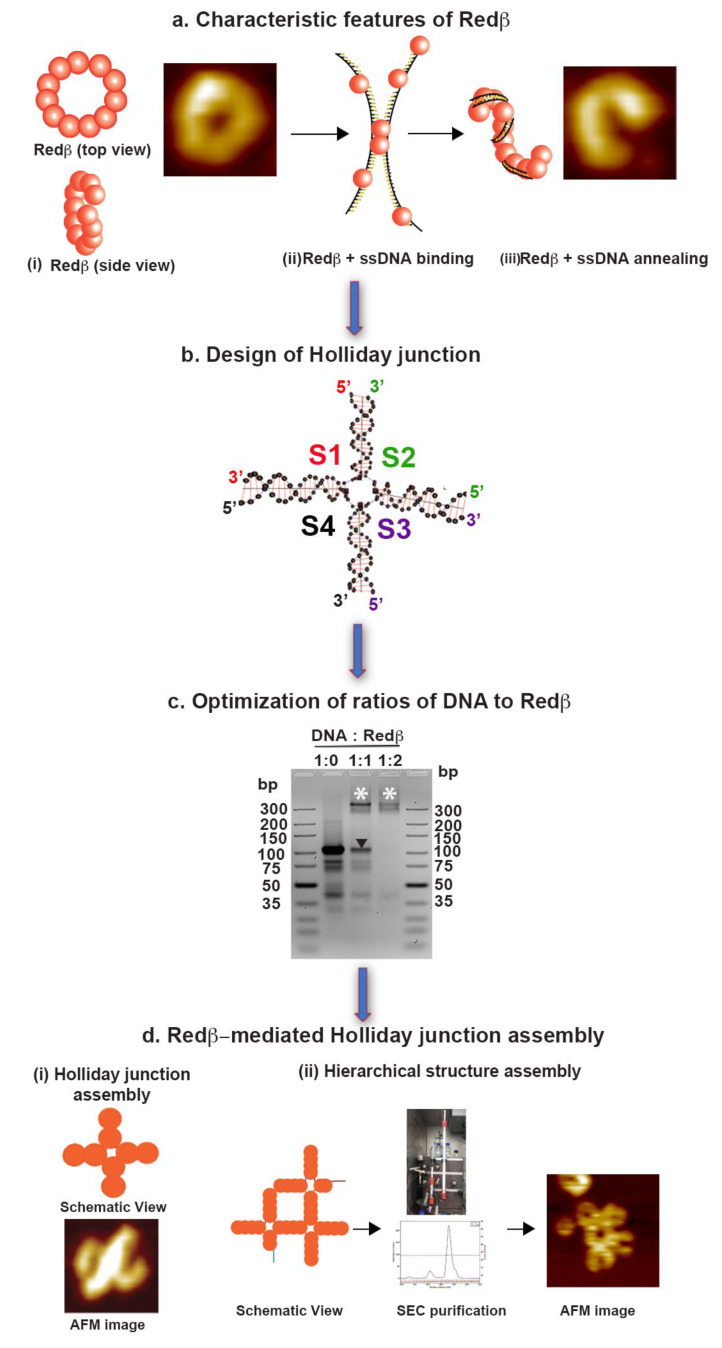
Experimental strategy for the Redβ-mediated assembly of Holliday junction (HJ) structures. (**a**) Characteristic features of Redβ. Models of the Redβ quaternary structure are shown for the protein alone (20 × 20 nm^2^), in complex with ssDNA, and in an annealed complex in the presence of two complementary ssDNA (20 × 20 nm^2^). The models were adapted from [29]. (**b**) Design of HJ structures. Different HJ structures composed of four ssDNA strands (S1–S4) were designed using UNIQUEMER 3D [41] and employed as substrates in this study. (**c**) Optimization of DNA/Redβ ratios by gel electrophoresis. The arrowhead pointing downwards indicates the free ssDNA, while the asterisks indicate the assembled HJ structures. (**d**) HJ assembly by Redβ. (**i**) Schematic view and atomic force microscopy (AFM) image of a single HJ structure (50 × 50 nm^2^). (**ii**) Schematic view, size exclusion chromatography (SEC) purification, and AFM image of a hierarchical HJ assembly (100 × 100 nm^2^).

Many phages encode their own homologous recombination systems often as 5′-3′ exonucleases paired with single-strand annealing proteins (SSAPs). In the lambda phage, the *red* recombination system includes lambda exonuclease (Redα) paired with the SSAP Redβ [27]. Genome integrity is maintained by repair of double-strand breaks (DSB) through homology directed repair (HDR) [28]. One of the reaction pathways of HDR involves single-strand annealing (SSA) mediated by SSAPs. SSAPs have been divided into two categories, namely ATP-dependent, e.g., RecA and Rad51, and ATP-independent, which in particular includes Redβ [29]. Redβ is a 30 kDa protein and a central player in the recombinant DNA engineering method called recombineering [30,31,32]. In the absence of DNA, Redβ polymerizes to form a shallow right-handed helix. Upon annealing two DNA strands, Redβ forms a very stable left-handed helical nucleoprotein filament (Figure 1a). These filaments have a curved form without rigid edges [29]. Optical tweezer single-molecule studies revealed that the Redβ nucleoprotein filament is nucleated by a remarkably stable DNA clamp to initiate HDR [33], while biochemical studies have established a complete model of HDR [34]. In this work, we utilized the annealing efficiency of Redβ to coat single-stranded (ss) DNA with Redβ monomers and then anneal them to complementary sequences to form DNA nanostructures based on rigid HJ motifs. The aim of this work was to (i) assess the potential of Redβ in the in vitro assembly of complex DNA structures, (ii) investigate whether the protein-coated DNA nanostructures provide a viable route for crystallizing the DNA–protein complexes, and (iii) produce extended lattices of rigid DNA–protein nanostructures at room temperature for applications in nanoelectronics [35,36], plasmonics [37,38], and molecular lithography [39,40].

Few previous attempts to employ proteins in the construction of DNA nanostructures have been reported. For instance, Praetorius et al. made efforts to synthesize a complete DNA origami nanostructure through stitching complementary sequences by DNA-binding TAL proteins [42]. The TAL proteins not only annealed but also provided rigidity to the DNA origami structures. Similarly, Schiffels et al., demonstrated the stiffening of flexible dsDNA structures using the HDR protein RecA [43]. In contrast to these proteins, however, Redβ does not form linear protein–DNA filaments but rather ring-like structures in the presence and absence of complementary ssDNA (see Figure 1a). While this intrinsic curvature may impose some limitations to nanostructure assembly, it may also present a possibility to synthesize more complex shapes and structures. Upon ssDNA binding, Redβ breaks into different nucleoprotein filaments depending on the length of the ssDNA (see Figure 1a) [29]. When a complementary ssDNA sequence is added, a highly stable dsDNA–protein complex is formed. Since it does not require ATP to anneal, this Redβ-assisted duplex formation occurs already at room temperature. During annealing, every Redβ protein monomer occupies approximately 11 bases in the ssDNA sequence, and efficient DNA annealing requires at least 15–20 base pairs [29]. Therefore, we have designed different four-arm HJ structures from 44 mer, 66 mer, and 88 mer oligonucleotides (Figure 1b). After determining the optimal ssDNA/Redβ ratio for Redβ-assisted annealing (Figure 1c), the HJ structures were assembled at room temperature and characterized by atomic force microscopy (AFM, Figure 1d). Furthermore, we also have synthesized hierarchical assemblies of HJ structures (Figure 1d) in order to assess the ability of Redβ to form complex and extended DNA nanostructures and pave the way for its inclusion in the DNA nanotechnology toolkit.

## 2. Results and Discussion

### 2.1. Assembly of Blunt-End HJ Structures from 44 mer, 66 mer, and 88 mer Oligonucleotides at 37 °C

**Figure 2:**
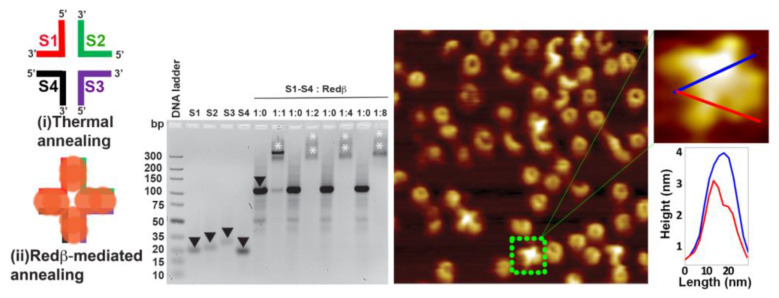
The schematic structures show the 44 mer HJ junction design assembled by thermal (**i**) and Redβ-mediated (**ii**) annealing. The sodium borate agarose gel analysis shows the saturation concentration ratio of Redβ to ssDNA. The downward-facing arrowheads (black) in lanes S1, S2, S3, and S4 of the gel image indicate the free ssDNA, while the asterisks indicate the HJ structures. The black downward-facing arrowhead in the first lane 1:0 indicates self-annealed HJ structures without protein. Note that the apparent differences in HJ band intensity for the different ssDNA/Redβ ratios are not due to any loss of DNA but rather due to a smearing of the bands (indicated by further asterisks), which results from the formation of a variety of different DNA–protein complexes. The AFM image depicts the resulting Redβ-mediated HJ assemblies. The height profiles were taken along the lines indicated in the zoom. The AFM image has a size of 300 × 300 nm^2^ and a height scale of 4 nm.

In a 44 mer HJ structure, every arm has a 22 mer dsDNA sequence. In order to estimate the optimal ssDNA/Redβ ratio during Redβ-assisted assembly, different ratios ranging from 1:1 to 1:4 were tested. For this, the individual strands S1–S4 were first coated with Redβ at 37 °C, subsequently mixed together, and incubated at 37 °C in order to anneal the HJ structure. Interestingly, even for the DNA mixture without Redβ, a significant shift of the corresponding band is observed in the gel image in Figure 2. This indicates that at this temperature, there is already some hybridization of the four partially complementary ssDNAs occurring. In the presence of Redβ, however, a much stronger shift is observed. In particular, at an ssDNA/Redβ ratio of 1:1, the agarose gel analysis shown in Figure 2 reveals a broad band corresponding to the ssDNA completely coated with Redβ monomers with some fast-migrating bands corresponding to residual ssDNA sequences with incomplete Redβ coating. Increasing the Redβ concentration resulted in all ssDNA sequences being coated with no residual ssDNA detected. The gel bands have a smeared look due to the weak interaction of ssDNA with Redβ. From these initial tests, we have decided to use 1:1 and 1:2 ratios of ssDNA/Redβ to achieve complete coating of the oligonucleotides and promote HJ assembly. After incubating the individual ssDNA strands with Redβ, all four sequences were mixed to form the HJ structure at room temperature. However, the AFM image of the assembled HJ structures in Figure 2 shows plenty of full rings and few C-shaped structures corresponding to free Redβ and incomplete HJ structures even at an ssDNA/Redβ ratio of 1:2. This mixture of structures further supports the weak binding of Redβ to short ssDNA sequences. Nevertheless, a few four-arm HJ structures can be identified in the AFM image in Figure 2 and are in fair agreement with the design (see the schematic structure in Figure 2). Since each arm can accommodate about two monomers, each of them exhibits a tiny protrusion. Typically, the ring-like structure formed by the Redβ monomers has a height of approximately 3 nm [29], which is in good agreement with height of the HJ arms as observed in the height profile in Figure 2. Furthermore, patches of increased height are visible in the center of the four-arm HJ structure due to the crossover of the protein–DNA filaments.

**Figure 3:**
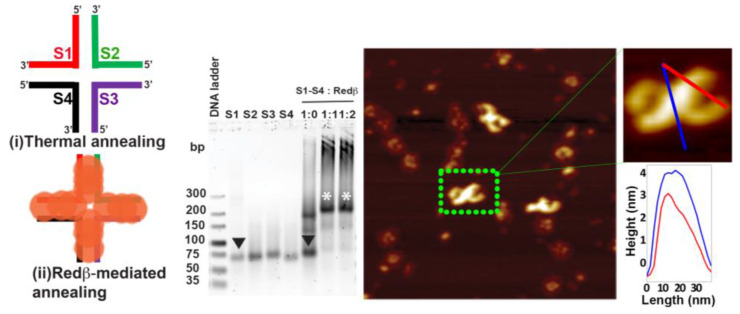
The schematic structures show the 66 mer HJ junction design assembled by thermal (**i**) and Redβ-mediated (**ii**) annealing. The sodium borate agarose gel analysis shows the saturation concentration ratio of Redβ to ssDNA. The downward-facing arrowheads (black) in the gel image indicate the free ssDNA, while the white asterisks indicate the HJ structures. The AFM image depicts the resulting Redβ-mediated HJ assemblies. The height profiles were taken along the lines indicated in the zoom. The AFM image has a size of 300 × 300 nm^2^ and a height scale of 4 nm.

The 66 mer ssDNA sequence was tested with ssDNA/Redβ ratios of 1:1 and 1:2. The broader bands of the Redβ–ssDNA complex observed in Figure 3 may indicate a slightly increased affinity of Redβ to the longer ssDNA sequence. Unlike the case of the 44 mer oligonucleotide, the gel analysis in Figure 3 shows that the 66 mer sequence has been completely coated with Redβ already at an ssDNA/protein ratio of 1:1. The AFM image in Figure 3 shows the 66 mer HJ structures assembled at this ratio. No full ring structures corresponding to Redβ monomers assembled without DNA are observed in this sample, which is thus indicative of the complete saturation of the ssDNA sequences. The 66 mer HJ structures are composed of two half-ring shapes connected at the central crossover point. Since each 33 bp arm can accommodate about three Redβ monomers, a total of six monomers are present on one half of the HJ structure. It is known from the literature [29] that a complete Redβ ring is composed of 11–12 monomers. In agreement with that, the AFM image in Figure 3 shows that the six monomers on each side of the crossover point of the HJ form a half-ring, resulting from the natural curvature of the Redβ filament, which bends the dsDNA arms of the HJ structures upon addition of a third monomer.

**Figure 4:**
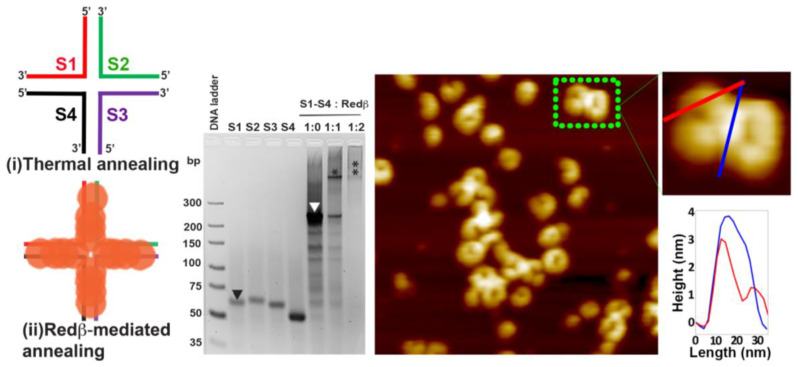
The schematic structures show the 88 mer HJ junction design assembled by thermal (**i**) and Redβ-mediated (**ii**) annealing. The sodium borate agarose gel analysis shows the saturation concentration ratio of Redβ to ssDNA. The downward-facing black and white arrowheads in the gel image indicate the free ssDNA and self-annealed HJ structures without protein, respectively, while the asterisks indicate the protein-annealed HJ structures. The AFM image depicts the resulting Redβ-mediated HJ assemblies. The height profiles were taken along the lines indicated in the zoom. The AFM image has a size of 300 × 300 nm^2^ and a height scale of 4 nm.

The 88 mer oligonucleotides were also incubated with ssDNA/Redβ ratios of 1:1 and 1:2. As can be seen in the gel analysis in Figure 4, the ssDNA sequences are not completely coated at the 1:1 ratio, which can be attributed to the larger number of available Redβ-binding sites in the longer ssDNA strands. At this ratio, a large fraction of free ssDNA is observed in the form of fast-migrating bands. At an ssDNA/Redβ ratio of 1:2, however, complete saturation of ssDNA with Redβ monomers is observed with the corresponding band migrating much slower. Moreover, the completely saturated band at the 1:2 ratio looks very faint compared to the case of the 66 mer oligonucleotide (see Figure 3 and Figure 4). These observations indicate that the interaction affinity of Redβ and ssDNA weakens once the ssDNA sequence exceeds a certain length. In the AFM image of the assembled 88 mer HJ structures in Figure 4, there are many full rings and plenty of half-rings observed at an ssDNA/Redβ ratio of 1:1. Due to their long sequence length, it may be possible for the 88 mer HJ structures to show partially and completely closed rings on both sides of the crossover point. There are about four monomers bound on each 44 bp arm, corresponding to 1/3 of the complete ring shape. When the four monomers in each of the four arms come together, their intrinsic curvature yields a HJ structure composed of two almost ring-like shapes with a prominent bulge at the crossover point.

Lilley described two broad classes of HJ conformations, namely open and stacked [44]. The open conformation has a characteristic feature of four arms being at a 90° angle to each other with a lack of central base pairing. The stacked form has a characteristic feature of slow branch migration as shown by several studies [5,6,45] and is characteristic for the HJ in the absence of proteins. Redβ, however, adopts a characteristic right-handed helix in the absence of DNA and changes its helicity to being left-handed upon ssDNA annealing (see Figure 1) [29], with the characteristic C shapes of the DNA–protein filaments indicating strong DNA bending. Based on our current understanding, this Redβ-induced bending of the DNA strands will probably affect the conformation of the assembled HJ, which may thus deviate from the known isomers described above. However, further crystallographic or numerical studies will be required to elucidate the detailed DNA conformations of the different Redβ-mediated HJ structures.

### 2.2. Hierarchical Assembly of HJ Structures with Sticky Ends

**Figure 5:**
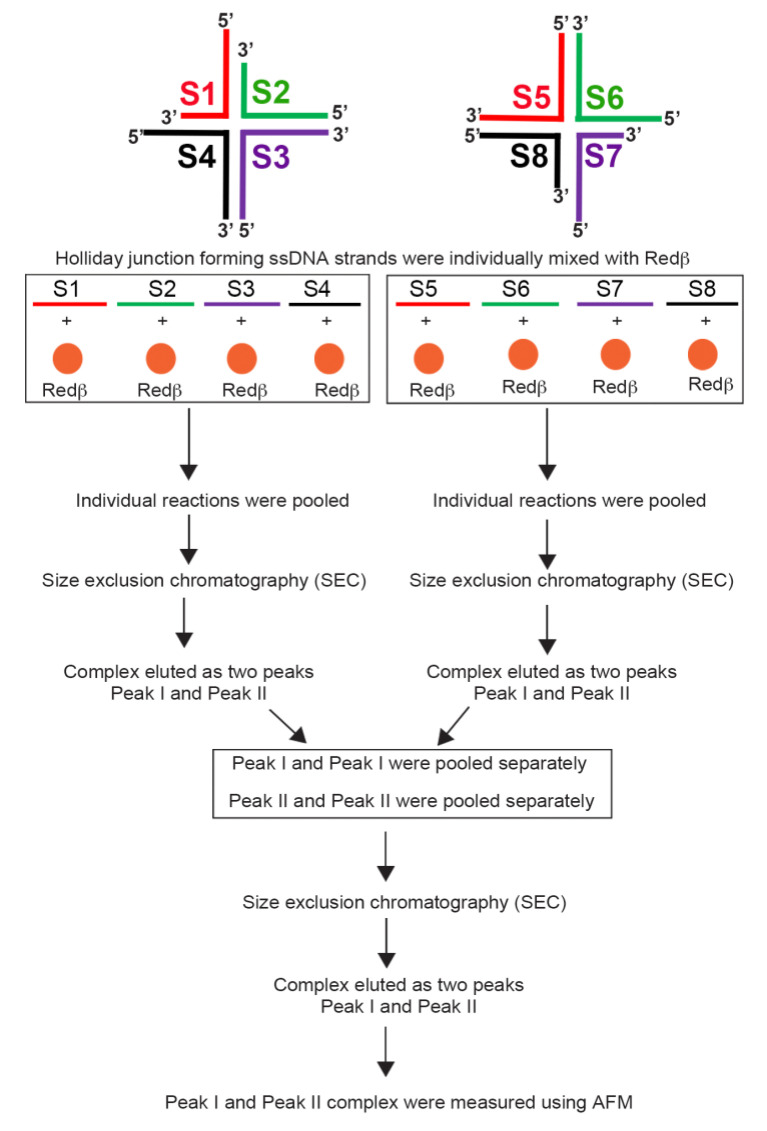
Experimental strategy for species enrichment of S1S4 and S5S8 HJ structures and purification of final hierarchical assemblies using SEC.

We have further designed HJ structures with sticky ends for hierarchical assembly. The experimental strategy for hierarchical self-assembly is outlined in Figure 5. For the purification of the single HJ structures and hierarchical assembly, the amount of Redβ has to be scaled up in order to meet the detection limit of the SEC purification methodology. For this, we have chosen an ssDNA/Redβ ratio of 1:25 based on our experimental results when 88 mer were purified by SEC (see Supplementary Materials).

**Figure 6:**
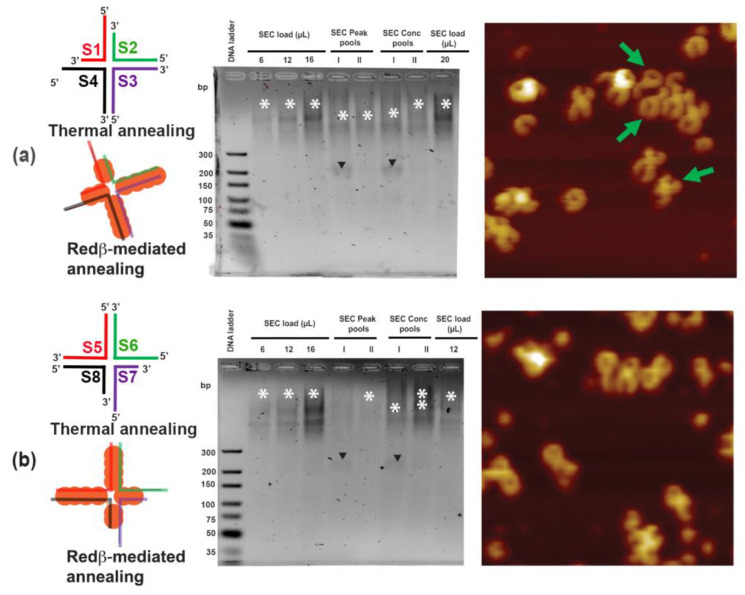
The schematic structures show the (**a**) S1S4 and (**b**) S5S8 HJ junction designs assembled by thermal and Redβ-mediated annealing. The corresponding sodium borate agarose gel analyses were conducted with the input and the pooled fractions after SEC, as explained in Figure 5. The downward-facing arrowheads in the gel image indicate the free ssDNA, while the asterisks indicate the HJ structures. The AFM images of Peak I (see Figure 5) were recorded after SEC purification. The AFM images show completely formed HJ structures with the green arrows indicating HJ structures with one arm missing. The AFM images have a size of 300 × 300 nm^2^ and a height scale of 4 nm.

The schematic structure in Figure 6a shows that two arms (S2S3 and S3S4) of the S1S4 HJ structure are ds 44 mer, which can accommodate about four Redβ monomers. The other two arms (S4S1 and S1S2) are each composed of two domains: a ds 22 mer close to the crossover point and an ss 22 mer at the distal end of the arm, which is acting as a sticky end. A similar design is also employed for the S5S8 HJ structure (Figure 6b). In the S1S4 HJ structure, the sticky ends of the S4S1 and S1S2 arms are complementary to those of the S6S7 and S7S8 arms of the S5S8 HJ structure, respectively. Interestingly, we observed efficient HJ structure assembly by Redβ at (23 ± 2) °C. Therefore, from here on, we performed HJ assembly as well as hierarchical assembly using Redβ at 23 °C. The individual Redβ-assembled S1S4 and S5S8 HJ structures were subjected to SEC for enrichment of the individual species (see Figure 5 and Materials and Methods). The enriched species from the two peaks obtained in SEC and fractions from each peak’s summit were pooled and analyzed along with load and concentrated samples by agarose gel electrophoresis (see Figure 6). For the S1S4 and S5S8 designs, the SEC loads show the presence of HJ species (see asterisks in the corresponding lanes in Figure 6). Pooled and concentrated Peak I fractions show the presence of free DNA substrates (black arrowheads), while the Peak II samples show complete saturation (asterisks). The AFM image of the S1S4 HJ structures in Figure 6a shows highly concentrated single species with sticky ends at two of the arms. Since the S2S3 and S3S4 arms have lengths of 44 nucleotides, many S1S4 HJ structures have an almost closed ring at one side and a half-ring at the other. Nevertheless, a certain variability of HJ structures is observed and can be attributed to the presence of different numbers of Redβ monomers. Upon binding with ssDNA, the Redβ monomers adhere and grow filaments of multiple lengths. Therefore, an 88 mer ssDNA does not necessarily have to be occupied by precisely eight Redβ monomers. If there are only seven monomers on an 88 mer ssDNA instead of eight, the HJ structure will exhibit a slightly different length and curvature. For instance, there are HJ structures observed with one arm being shorter than the others or even missing completely (highlighted by green arrows in Figure 6), which demonstrates the necessity of enriching the individual species. Even though the individual species of S1S4 and S5S8 were enriched by SEC, there are still plenty of incomplete HJ structures observed in the AFM images in Figure 6 that have only one, two, or three arms.

**Figure 7:**
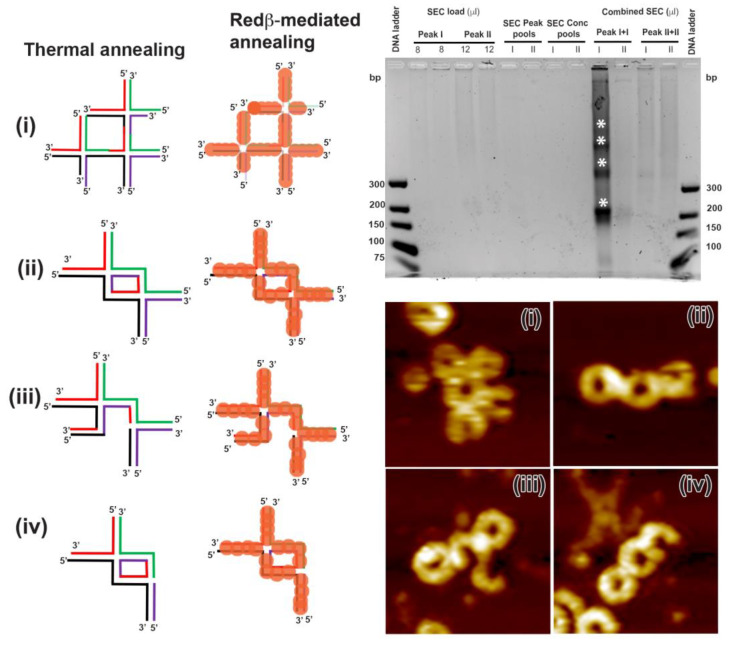
The schematic structures show possible hierarchical assemblies composed of S1S4 and S5S8 HJ junction structures assembled by thermal and Redβ-mediated annealing. The corresponding sodium borate agarose gel analysis was conducted with the input and the pooled fractions after SEC, as explained in Figure 5. The asterisks indicate the hierarchical HJ assemblies. The AFM images of Peak I (see Figure 5) were recorded after SEC purification and show hierarchical HJ assemblies corresponding to the schematic structures (**i**–**iv**). The AFM images have a size of 125 × 125 nm^2^ and a height scale of 4 nm.

For hierarchical assembly, Peak I and Peak II from the S1S4 assembly were mixed with the corresponding Peak I and Peak II from the S5S8 assembly, respectively. Four discrete bands can be seen in the lane corresponding to the concentrated Peak I of S1S4 and S5S8 HJ structures after SEC (gel image in Figure 7, white asterisks). Surprisingly, the Peak II combination did not show any hierarchical assemblies (see Figure 7). Therefore, the Peak I sample was used for AFM imaging. Several different structures were observed by AFM (see Figure S2), a selection of which is shown in Figure 7. Both the S1S4 and S5S8 HJ structures have shapes consisting of a closed ring connected to a half-ring due to the differences in the dsDNA domains in the different arms. When one S1S4 and one S5S8 anneal with each other, however, they form structure (ii), which resembles a chain of three closed rings (see Figure 7). There are plenty of such chain-like structures observed in the AFM images in Figure S2. In some structures, however, individual arms are missing, or additional arms are present (see Figure 7, structures (iii) and (iv)). Interestingly, if one of the sticky arms of the S1S4–S5S8 dimer is free and anneals with a complementary ssDNA–Redβ complex, it will form a 2D square lattice structure (i) as seen in the corresponding AFM image in Figure 7. Formation of multiple-ring structures is also possible due to a cross-linking of free sticky arms with hierarchical structures. In summary, any free S1S4 and S5S8 structure can anneal with any complementary sticky end, as observed after SEC purification in the AFM images in Figure 7. Therefore, we next attempted to assemble individual 66 mer HJ structures into extended hierarchical networks.

### 2.3. Assembly of Hierarchical Networks Composed of 66 mer HJ Structures

**Figure 8:**
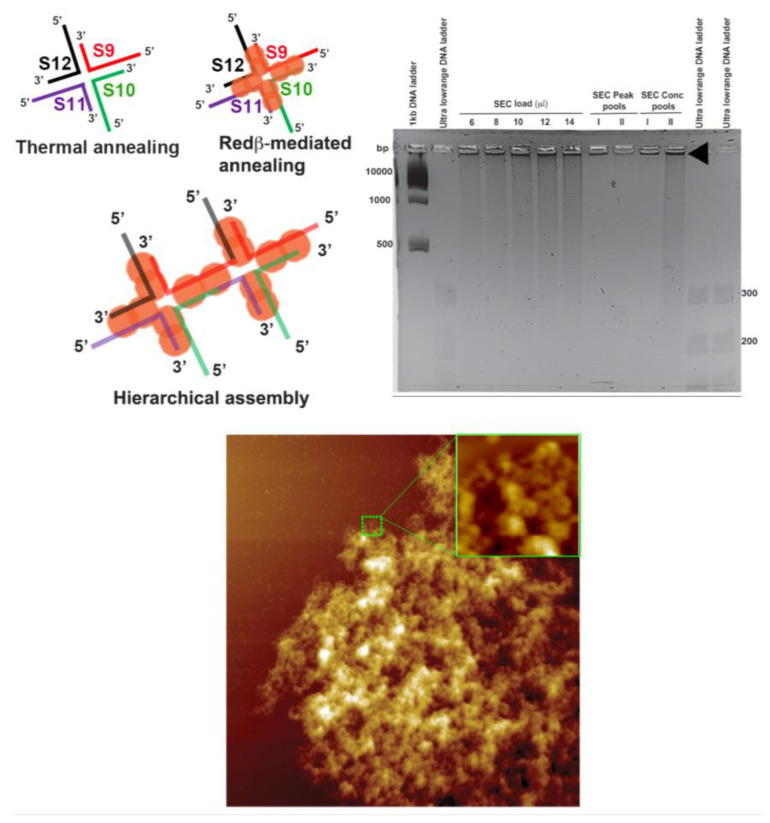
The schematic structures show the design and hierarchical assembly of a 66 mer HJ structure in which each arm has complementary sticky end sequences for hierarchical assembly. The sodium borate agarose gel analysis shows that the assembled structures are too large to enter the gel (indicated by the black arrowhead). The AFM image of the concentrated Peak I shows the efficient Redβ-assisted hierarchical assembly of the 66 mer HJ structures. A zoomed section in the AFM image depicts the microstructures present inside the hierarchical structure. The AFM image has a size of 5 × 5 µm^2^ and a height scale of 20 nm.

We aimed at a two-dimensional hierarchical structure assembly by designing a 66 mer HJ structure with self-complementary arms in its structure. In this HJ structure, each arm is composed of a 22 mer ds domain and a 22 mer sticky end. The sticky end of arm S12S9 is complementary to that of arm S10S11, whereas the sticky end of arm S9S10 is complementary to that of arm S11S12 (see the schematic structures in Figure 8). Individual sequences were first treated with Redβ for annealing and subjected to SEC (see Materials and Methods) followed by agarose gel electrophoresis to analyze the two peak fractions. However, we found that the concentrated Peak I and Peak II fractions contained structures that were too large to enter the gel even after a long run (see gel analysis in Figure 8, arrowhead). The rapid Redβ-mediated assembly of large complex structures is also evident from the AFM image in Figure 8. The four sticky arms of the HJ structures were annealed with self-complementary arms in all the directions, thus forming a huge clump. When analyzed carefully, the clump has numerous highly branched microstructures (see AFM zoom in Figure 8). Since the dsDNA–Redβ complex can exhibit highly flexible structural conformations in all directions, the polymerization reaction extended in all directions until the free sticky ends were depleted. The sizes of the clumps observed in the AFM images were thus of the order of several microns. Such mesh-like structures may be interesting for the crystallographic investigation of Redβ–dsDNA complexes and thereby provide a better biophysical understanding of recombineering. Moreover, this extraordinary ability of Redβ to anneal complex dsDNA assemblies at room temperature may open up possible applications of Redβ and other SSAPs in DNA nanotechnology.

## 3. Materials and Methods

### 3.1. Protein Expression, Affinity, and Preparative Gel Filtration Chromatography

For in vitro studies, Redβ was purified using one-step Strep-tag II/Strep-Tactin affinity chromatography which allows mild protein purification under physiological conditions as described previously [29,34,46]. Quality and quantities of the protein from the two steps above were monitored by analytical SDS-PAGE analysis. Purified Strep-tag II-tagged protein was >95% pure at a concentration range of 0.5–10.0 mg/mL when quantified on a Nanodrop 1000 (Thermo Fisher Scientific GmbH, Bremen, Germany).

### 3.2. Establishment of a Holliday Junction Assembly Protocol Using Redβ

Different immobile HJ structures with different lengths were designed using UNIQUEMER 3D software version 1.0 [41]. Oligonucleotides were purchased from Metabion international AG (Planegg/Steinkirchen, Germany) (HPLC-purified). To optimize HJ assembly, 10 pmol of each ssDNA was incubated with 10, 20, 40, or 80 pmol of Redβ at 37 °C for 20 min in four sterile 1.5 mL Eppendorf tubes. After this step, all reaction contents from respective ratios were mixed in one sterile 1.5 mL Eppendorf tube (final volume 20 μL) and incubated at 37 °C for 20 min. The reaction was stopped by adding 4 µL of 6 using 1× sodium borate buffer DNA loading dye and 3 µL of 50% glycerol. As controls, each individual ssDNA was also loaded. The reaction mixtures were loaded and electrophoresed on 2% or 3% sodium borate agarose gels using 1× sodium borate buffer. After electrophoresis, gels were stained with 1× sybrGold (Thermo Fisher Scientific GmbH, Bremen, Germany) in 1× sodium borate buffer for 15 min at 25 °C. Visualization of DNA was carried out using the Gel DocTM XR+ imaging system (Bio-Rad laboratories GmbH, Feldkirchen, Germany). All the sequences are listed in Table S1.

For AFM imaging of 44 mer, 66 mer, and 88 mer HJ structures assembled at room temperature (see Figure 1, Figure 2 and Figure 3), 1:4 ratios (in µM) of individual ssDNA sequences and Redβ were mixed in separate Eppendorf tubes and incubated at 37 °C for 20 min. Then, the complete volume of all the ssDNA–protein complexes was added to a sterile Eppendorf tube, mixed well, and incubated at 37 °C for 20 min to facilitate HJ structure formation. The same protocol was used for the hierarchical structure assembly without SEC purification and AFM analysis at 5 and 24 h time intervals (see Figure S3). The AFM imaging of the HJ structures and hierarchical assembly are described in Section 3.5.

### 3.3. Redβ-Mediated Annealing and Purification of Hierarchical HJ Assemblies with Sticky Ends (S1S4 and S5S8)

For S1S4 + Redβ, 12.5 µL of 66 mer ssDNA S1, S2 and 88 mer ssDNA S3, S4 (1 µM) was pipetted into four individual sterile 1.5 mL Eppendorf tubes. For S5S8 + Redβ, 12.5 µL of 88 mer ssDNA S5, S6 and 66 mer ssDNA S7, S8 (1 µM) was pipetted into four individual sterile 1.5 mL Eppendorf tubes. An amount of 370 µL of 87 µM Redβ (pre-incubated at 37 °C for 20 min) was pipetted into each of these 8 tubes and the final volumes were adjusted to 1250 µL by addition of 870 µL of sterile B1 buffer (50 mM Tris-HCl pH 8.0, 150 mM NaCl, and 1 mM MgCl_2_). The reaction contents were mixed well by gentle pipetting and incubated at 25 °C for 20 min. Afterwards, the contents of all four tubes, namely S1, S2, S3, and S4, were mixed in a 15 mL Falcon tube and incubated overnight at 25 °C. Similarly, the contents of the other four tubes, namely S5, S6, S7, and S8, were mixed in another 15 mL Falcon tube and incubated overnight at 25 °C. Following overnight incubation, the reaction mixture S1S4 + Redβ was loaded using a 5 mL sample loop onto the preparative superdex 200 column (16/600) (GE Lifesciences, Chicago, IL, USA) connected to an Äkta purifier (GE Healthcare, Chicago, IL, USA). The column was pre-equilibrated with B2 buffer (100 mM Tris-HCl pH 8.0, 150 mM NaCl, and 1 mM MgCl_2_). Preparative size exclusion chromatography (SEC) was carried out at 1 mL/min flowrate. The eluting fractions were collected and based on the profile of the peaks, and only the summit fractions from the peaks were analyzed and pooled. A similar protocol was followed for the reaction mixture S5S8 + Redβ as well. The eluting fractions were collected and pooled as described above. For the purpose of quality control, we performed purification of S1S4 + Redβ species as well as S5S8+Redβ species separately as the first step. Once we confirmed the purification of the desired species, we went further and combined S1S4 + Redβ with the S5S8 + Redβ species. Peak I pools and Peak II pools of S1S4 + Redβ with S5S8 + Redβ runs were combined appropriately and purified further by another preparative SEC run. The resulting peak fractions were grouped as Peaks I and II. Purified complexes were analyzed by agarose gel electrophoresis in order to visualize DNA and on 8% native PAGE or 4–16% gradient BlueNative PAGE gels followed by colloidal coomassie staining to visualize the protein. Afterwards, the desired species were used for AFM imaging.

### 3.4. Redβ-Mediated Assembly of Two-Dimensional Hierarchical HJ Networks (S9S12)

For the two-dimensional hierarchical network assembly, 4 sticky-end ssDNAs (66 nucleotides long), namely S9, S10, S11, and S12, which could assemble in a left–right and up–down manner (see Figure 8) were used along with Redβ. We set up an optimization protocol to check the Holliday junction network formation by Redβ similar to the S1S4 + Redβ and S5S8 + Redβ work above. SEC purification (see Figure 5) was used for enriching the S9S12 + Redβ species. Following SEC, peak fractions were pooled and analyzed on 2% sodium borate agarose gel to visualize the S9S12 DNA and S9S12 + Redβ species. We used 8% native PAGE gel followed by colloidal coomassie staining to visualize the protein present in the S9S12 + Redβ complex. Afterwards, the desired species from Peak I was used for AFM imaging.

### 3.5. AFM Imaging

An amount of 5 µL of the Redβ-annealed HJ assemblies was deposited onto freshly cleaved mica surfaces. An amount of 100 µL of fresh B2 buffer was added and the samples were allowed to adsorb for 15 min. The samples were washed with 5 mL of HPLC-grade water and dried with a stream of ultrapure air. The AFM images were recorded in intermittent contact mode using an Agilent 5100 AFM (Agilent Technologies, Inc., Santa Clara, California, CA, USA) and HQ:NSC18/Al BS cantilevers (MikroMasch Europe, Wetzlar, Germany).

## 4. Conclusions

In this study, we have successfully optimized the conditions for the assembly of blunt- or sticky-ended HJ structures using Redβ. We have shown that these assembled structures are stable and hence could be purified by SEC. Our experiments with increasing lengths of HJ arms using three different lengths of ssDNA substrates support previous findings concerning the monomer spacing on DNA [29]. Most importantly, however, we have successfully demonstrated the ability of Redβ to assist in the concerted annealing of HJ structures and their hierarchical assembly into complex networks. As we have employed the full-length Redβ in this study, we observed left-handed helical filaments with curved ends. Future studies employing purified DNA binding domains or shorter DNA binding peptides of Redβ could aim at having more rigid-ended complex HJ assemblies. This could open up new avenues in the fields of macromolecular patterning and DNA nanotechnology.

## Supplementary Materials

The following are available online, Redβ-mediated annealing and purification of 88 mer HJ structures, gel analysis of the purified and enriched 88 mer HJ structures and corresponding AFM images, AFM images of hierarchical structures with SEC purification, AFM images of individual HJ structures and hierarchical structures without purification, oligonucleotide sequences.
