# Supplementary material for: Protein-Assisted Room-Temperature Assembly of Rigid, Immobile Holliday Junctions and Hierarchical DNA Nanostructures
Source: Molecules. 2020 Nov 3;25(21):5099. doi: 10.3390/molecules25215099 (PMC7663122; doi:10.3390/molecules25215099)
Supplement: Supplementary file 1 [file molecules-25-05099-s001.pdf]

Supplementary Materials

# Protein-Assisted Room-Temperature Assembly of Rigid, Immobile Holliday Junctions and Hierarchical DNA Nanostructures

Saminathan Ramakrishnan, Sivaraman Subramaniam, Charlotte Kielar, Guido Grundmeier, A. Francis Stewart, and Adrian Keller

Table of contents

1. Redβ-mediated annealing and purification of 88mer HJ structures.
2. Gel analysis of the purified and enriched 88mer HJ structures and corresponding AFM images.
3. Additional AFM images of hierarchical structures after SEC purification.
4. AFM images of individual 88 mer HJ structures and hierarchical structures without purification.
5. Oligonucleotide sequences.

## 1. Redβ-mediated annealing and purification of 88mer HJ assemblies.

12.5 µl of each 88mer ssDNA (1 µM) were pipetted into four individual sterile 1.5 ml Eppendorf tubes. 370 µl of 87 µM Redβ (pre-incubated at 37°C for 20 minutes) was pipetted into each tube and the final volumes were adjusted to 1250 µl by addition of 870 µl of sterile B1 buffer (50 mM Tris-HCl pH 8.0, 150 mM NaCl, and 1 mM $MgCl_2$). The reaction contents were mixed well by gentle pipetting and incubated at 25°C for 20 minutes. Afterwards, the contents of all four tubes were mixed in a 15 ml Falcon tube (Fisher scientific) and incubated overnight at 25°C. Following overnight incubation, the reaction mixture was loaded using a 5 ml sample loop onto the preparative superdex 200 column (16/600) (GE lifesciences) connected to Äkta purifier (GE healthcare). The column was pre-equilibrated with B2 buffer (100 mM Tris-HCl pH 8.0, 150 mM NaCl and 1 mM $MgCl_2$). Preparative size exclusion chromatography was carried out at 1 ml/minute flowrate. The eluting fractions were collected and based on the profile of the peaks, only the summit fractions from the peaks were analyzed and pooled. Purified Redβ-assembled HJs were analyzed by AFM imaging as well as on 8% native PAGE or 4-16% gradient BlueNative PAGE gels followed by colloidal coomassie staining to visualize protein. 2% sodium borate agarose gel electrophoresis was carried out in order to visualize DNA.

## 2. Gel analysis of the purified and enriched 88mer HJ structures and corresponding AFM images.

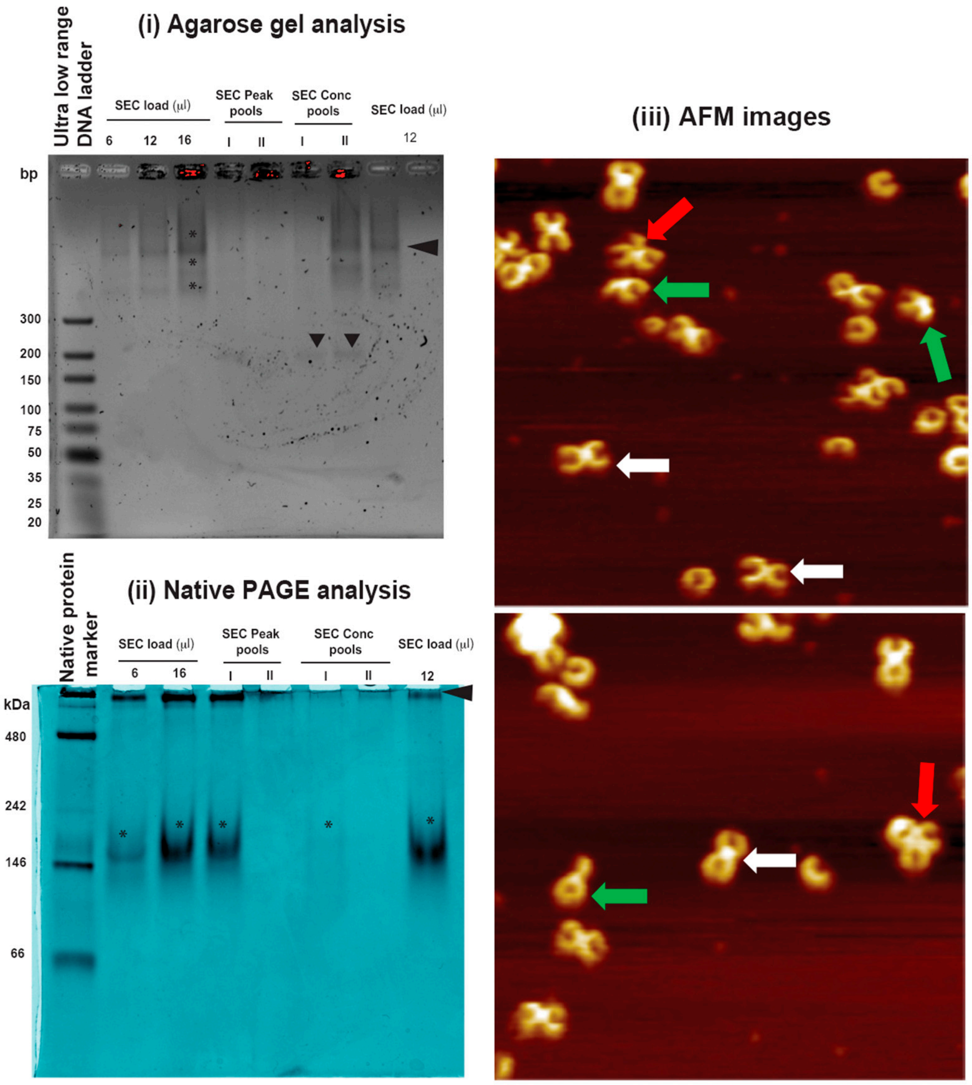


Figure S1. Purification and enrichment of single HJ species using SEC. (i) 2% sodium borate agarose analysis shows the concentrated HJ structures after purification using SEC. The asterisks (*) indicate the HJ structures. The lower arrowheads (faint bands) indicate the free DNA. The black sideway arrowhead depicts the HJ structures imaged by AFM. (ii) 8% native PAGE analysis shows the SEC purified HJ structures. The asterisks (*) and the black sideway arrowhead indicate different HJ structures. (iii) The AFM analysis of purified 88 mer HJ species shows completely formed structures (white arrows) as well as structures missing single arms (green arrows) and structures with additional arms (red arrows). The C-shaped structures represent DNA-protein filaments that did not participate in the annealing of complementary sequences and HJ formation. These individual C-shaped structures often interfere with other structures that are getting annealed and create HJ structures with multiple arms. The AFM images are 300 x 300 $nm^2$ in size and 4 nm in height.

## 3. Additional AFM images of hierarchical structures after SEC purification.

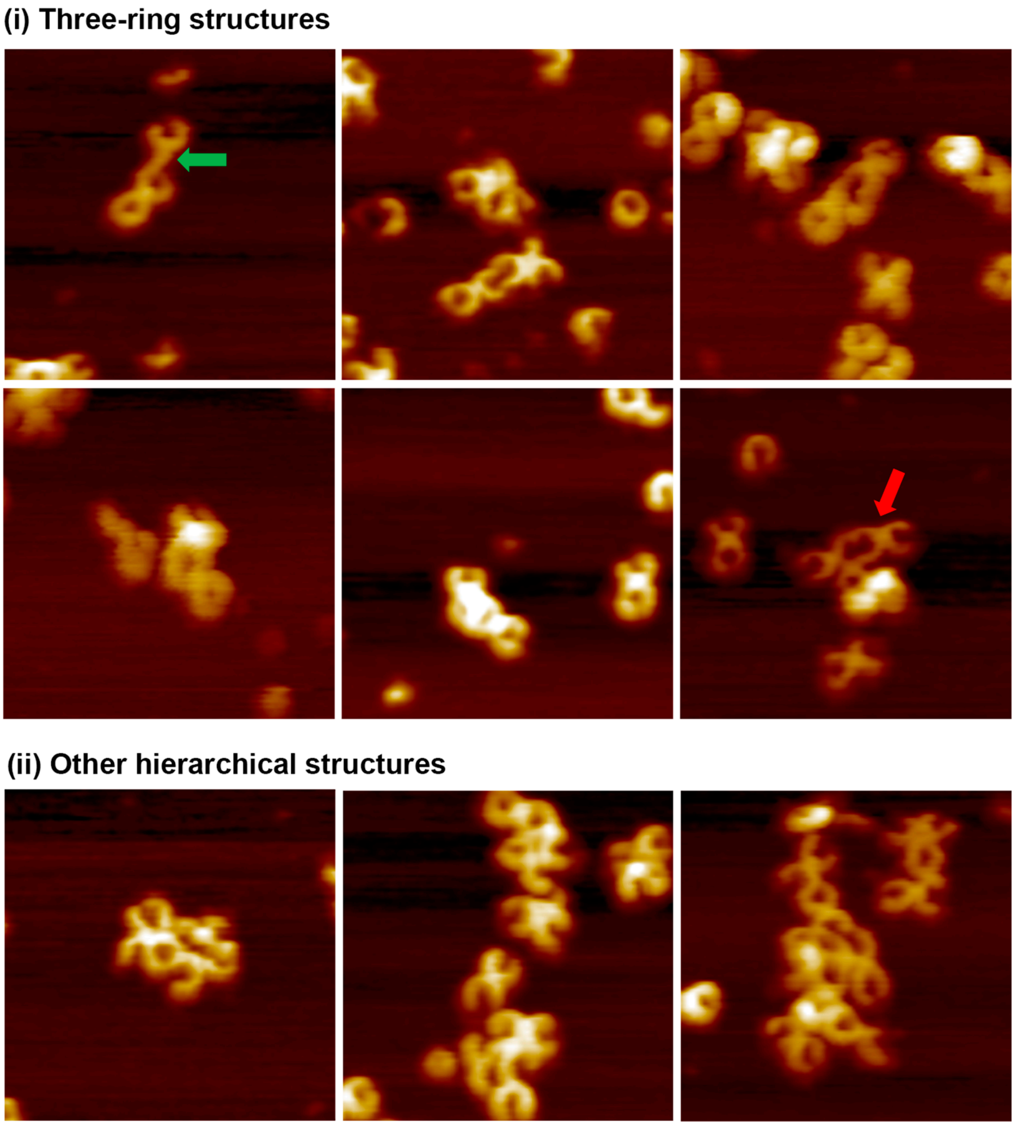


Figure S2. AFM images of concentrated and purified hierarchical structures after SEC. The AFM images show mainly two different structures, *i.e.*, (i) three-ring structures and (ii) irregular hierarchical structures. The three-ring structures are often observed with an arm missing (green arrow) or additional arms present (red arrow) due to non-specific annealing. The irregular hierarchical structures are the result of non-specific annealing of free C-shaped DNA-protein filament in the SEC pool. The examples are given to show the annealing efficiency of Redβ at room temperature. The AFM images are 200 x 200 $nm^2$ in size and 4 nm in height.

**4. AFM images of individual 88mer HJ structures and hierarchical structures without purification.**

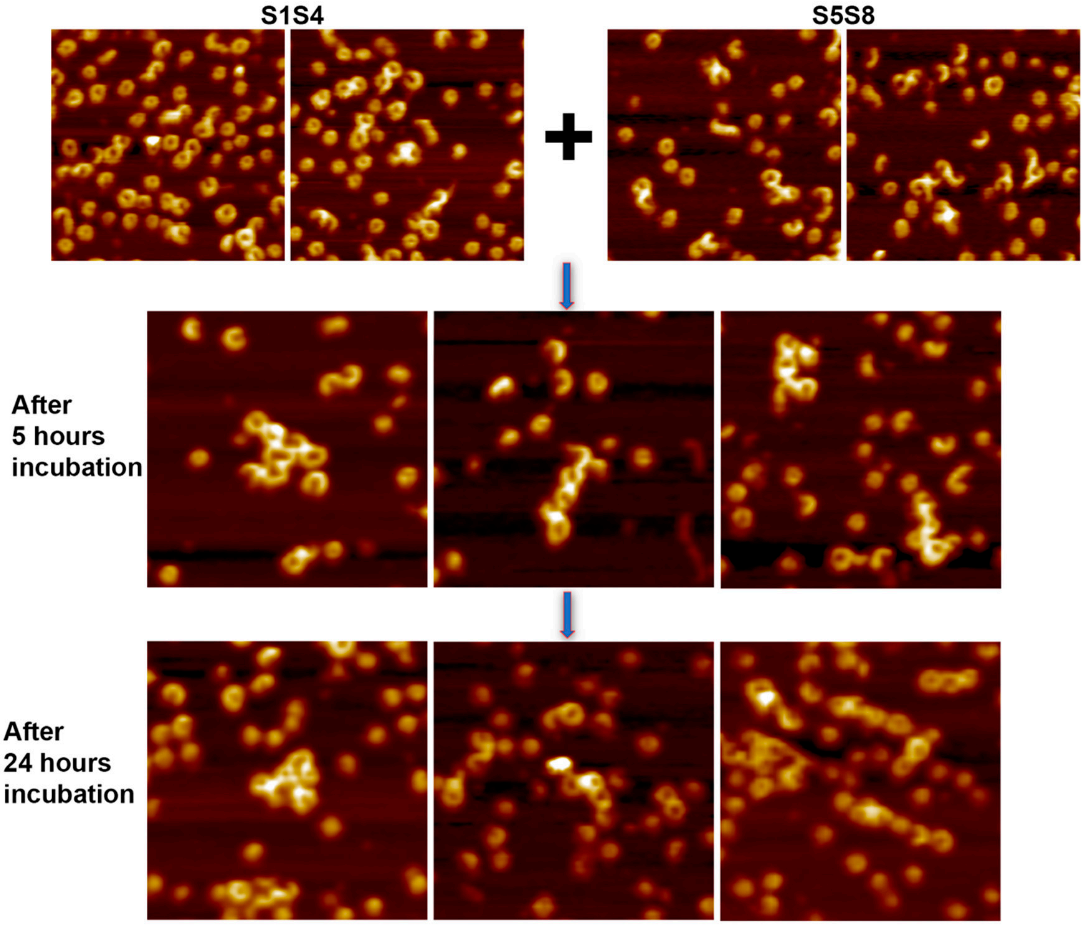


Figure S3. The AFM images of S1S4 and S5S8 show individual HJ structures with sticky ends, assembled in the one-pot annealing reaction without purification. Many completely formed HJ structures are observed but accompanied by additional Redβ rings and C-shaped DNA-protein filaments. The AFM images of hierarchical structures assembled after 5 hours and 24 hours reveal many irregular structures, which are the result of non-specific annealing of free C-shaped DNA-protein filaments in the pool. Purification and enrichment of individual HJ species are therefore necessary to remove the excess of Redβ rings and C-shaped filaments. The AFM images are 300 x 300 $nm^2$ in size and 4 nm in height.

## 5. Oligonucleotide sequences.

Table 1. ssDNA sequences used for 44mer HJ structure

| No. | Sequence (5′-3′) | GC (%) | Length | Identifier |
|---|---|---|---|---|
| 1 | CGATGCGAAATCTGTACGCGCACCACGTGAAGGTGCTAGTCCCC | 59 | 44 | S1 |
| 2 | GAATGTGTTGATGGTTAAGTTCTGCGCGTACAGATTTCGCATCG | 46 | 44 | S2 |
| 3 | GTGTCTATCATTAGCGGCTCAAGAACTTAACCATCAACACATTC | 41 | 44 | S3 |
| 4 | GGGGACTAGCACCTTCACGTGGTTGAGCCGCTAATGATAGACAC | 55 | 44 | S4 |

Table 2. ssDNA sequences used for 66mer HJ structure

| No. | Sequence (5′-3′) | GC (%) | Length | Identifier |
|---|---|---|---|---|
| 1 | ATGCCGCATACAACAAGTGGGGGGATGAATAGGATGTACTGCCT TTTTTATCGACTGTCCAATATG | 44 | 66 | S1 |
| 2 | CATATTGGACAGTCGATAAAAAAGGCAGTACATACTTGGTCCACT CTGGTACAAGATGAGGTGGAT | 42.43 | 66 | S2 |
| 3 | ATCCACCTCATCTTGTACCAGAGTGGACCAAGTAGTGCGCGATT GACGTTCGAGGCGGACTCTCCT | 55 | 66 | S3 |
| 4 | AGGAGAGTCCGCCTCGAACGTCAATCGCGCACTCCTATTCATCC CCCCACTTGTTGTATGCGGCAT | 56 | 66 | S4 |

Table 3. ssDNA sequences used for 88mer HJ structure

| No. | Sequence (5′-3′) | GC (%) | Length | Identifier |
|---|---|---|---|---|
| 1 | CAGTTCCCTAAGACATCGACGTAGGACCTTGATCAACGCAGCTAT GACTTCAGAGATTCTCTTATCTGGAAGCGTCCACGAACCGGCC | 52 | 88 | S1 |
| 2 | GGCCGGTTCGTGGACGCTTCCAGATAAGAGAATCTCTGAAGTCAT TTGTACACTTTTCTATTCACGAGCAAAGCCTGGTATACTAGAA | 45 | 88 | S2 |
| 3 | TTCTAGTATACCAGGCTTTGCTCGTGAATAGAAAAGTGTACAAACC CACATTGCCATCGGGCGAAATGGGGTGTGCAGCGCATGCTCG | 50 | 88 | S3 |
| 4 | CGAGCATGCGCTGCACACCCCATTTCGCCCGATGGCAATGTGGGT | 56.8 | 88 | S4 |

| | | | | |
|---|---|---|---|---|
| | AGCTGCGTTGATCAAGGTCCTACGTCGATGTCTTAGGGAACTG | | | |

Table 4. ssDNA sequences used for hierarchical structure assembly (S1S4)

| No. | Sequence (5′-3′) | GC (%) | Length | Identifier |
|---|---|---|---|---|
| 1 | GATTCCTTCCGGGCGAATTTACCCCAGTGAACGCTGGTCCCCGC GGTCATGCTGAAGGCGATAGTA | 58 | 66 | S1 |
| 2 | GCGGGGACCAGCGTTCACTGGGTTATTGGGTATGACGAGCTTAG ATTTGGTATATGGTCTCGACTA | 50 | 66 | S2 |
| 3 | TAGTCGAGACCATATACCAAATCTAAGCTCGTCATACCCAATAAG ATGTACGTTGGCGGACAGTTACATATTGTGCGCTGCCGGTGGC | 48 | 88 | S3 |
| 4 | GCCACCGGCAGCGCACAATATGTAACTGTCCGCCAACGTACATC GTAAATTCGCCCGGAAGGAATCCCGTAACGAGGCCACATTTTTG | 53 | 88 | S4 |

Table 5. ssDNA sequences used for hierarchical structure assembly (S5S8)

| No. | Sequence (5′-3′) | GC (%) | Length | Identifier |
|---|---|---|---|---|
| 1 | ATGTTGATCGGGAGAGGTCGAACCCTCTTTGATATTTGTGACTTT CGGGGCAACTATTTCCTCGGCGACCACGTTCGGTGACGGACCT | 52 | 88 | S5 |
| 2 | AGGTCCGTCACCGAACGTGGTCGCCGAGGAAATAGTTGCCCCGA CAGGAAGCTAACTAACATGAGACAAAAATGTGGCCTCGTTACGG | 53 | 88 | S6 |
| 3 | TCTCATGTTAGTTAGCTTCCTGACGCCTGGCCGTATCTTCGGCTT ACTATCGCCTTCAGCATGACC | 52 | 66 | S7 |
| 4 | AGCCGAAGATACGGCCAGGCGTAAGTCACAAATATCAAAGAGGG TTCGACCTCTCCCGATCAACAT | 50 | 66 | S8 |

Table 6. ssDNA sequences used for hierarchical structure assembly (S9S12)

| No. | Sequence (5′-3′) | GC (%) | Length | Identifier |
|---|---|---|---|---|
| 1 | CCAATACGTGCTCGCCGTCCAGCGACTCTTAATTGCGTTGGTGT TTTTGAGTGAGTCTCTAACCCG | 51 | 66 | S9 |
| 2 | CGCCAGTCGTCTATGGCGCTGACGGGTTAGAGACTCACTCAAAA AGGCGATGGTGAGCATGTCTGG | 56 | 66 | S10 |
| 3 | CTGGACGGCGAGCACGTATTGGCCAGACATGCTCACCATCGCCT ATCATAAGCGCACCCGCTGCCC | 62 | 66 | S11 |
| 4 | TCAGCGCCATAGACGACTGGCGGGGCAGCGGGTGCGCTTATGAT ACACCAACGCAATTAAGAGTCG | 58 | 66 | S12 |
